# Regional Gene Therapy for Bone Tissue Engineering: A Current Concepts Review

**DOI:** 10.3390/bioengineering12020120

**Published:** 2025-01-27

**Authors:** Matthew C. Gallo, Aura Elias, Julius Reynolds, Jacob R. Ball, Jay R. Lieberman

**Affiliations:** 1 Department of Orthopaedic Surgery, Keck School of Medicine of the University of Southern California, Los Angeles, CA 90033, USA; matthew.gallo@med.usc.edu (M.C.G.); amelias@usc.edu (A.E.); jr_125@usc.edu (J.R.); jacob.ball@med.usc.edu (J.R.B.); 2 Alfred E. Mann Department of Biomedical Engineering, Viterbi School of Engineering, University of Southern California, Los Angeles, CA 90089, USA

**Keywords:** gene therapy, tissue engineering, bone defect, bone graft, bone regeneration, BMP-2, osteoinductive, mesenchymal stem cells

## Abstract

The management of segmental bone defects presents a complex reconstruction challenge for orthopedic surgeons. Current treatment options are limited by efficacy across the spectrum of injury, morbidity, and cost. Regional gene therapy is a promising tissue engineering strategy for bone repair, as it allows for local implantation of nucleic acids or genetically modified cells to direct specific protein expression. In cell-based gene therapy approaches, a variety of different cell types have been described including mesenchymal stem cells (MSCs) derived from multiple sources—bone marrow, adipose, skeletal muscle, and umbilical cord tissue, among others. MSCs, in particular, have been well studied, as they serve as a source of osteoprogenitor cells in addition to providing a vehicle for transgene delivery. Furthermore, MSCs possess immunomodulatory properties, which may support the development of an allogeneic “off-the-shelf” gene therapy product. Identifying an optimal cell type is paramount to the successful clinical translation of cell-based gene therapy approaches. Here, we review current strategies for the management of segmental bone loss in orthopedic surgery, including bone grafting, bone graft substitutes, and operative techniques. We also highlight regional gene therapy as a tissue engineering strategy for bone repair, with a focus on cell types and cell sources suitable for this application.

## 1. Introduction

### Bone Loss—Definitions and Challenges

Segmental and critical-sized bone defects present significant challenges in orthopedic surgery, with substantial implications for patient outcomes and healthcare costs [1,2,3]. These defects occur in various clinical scenarios including high-energy trauma, fracture nonunion, spine pseudoarthrosis, revision total joint arthroplasty, tumor resection, and infection requiring debridement. Managing segmental bone defects with current treatments can incur healthcare costs as high as USD 300,000 per case and, despite technological and surgical advances, remains associated with significant patient morbidity and inconsistent outcomes [1].

Bone defects exist along a continuum, with distinct nomenclature based on defect size and injury characteristics. Critical-sized defects do not heal spontaneously and always require reconstructive intervention [2]. There is no single definition of what constitutes a critical-sized defect, as it is affected by defect geometry, location, patient factors (e.g., smoking status or the presence of medical comorbidities), and the integrity of the surrounding tissues. However, they are generally characterized as a defect length measuring 2–2.5 times the diameter of the affected bone, or circumferential bone loss greater than 50% for non-segmental defects [4,5].

The successful healing of bone defects depends on four essential elements: (1) an osteoconductive scaffold/matrix to support bone and vascular ingrowth, (2) osteoinductive growth factors that promote the mobilization and differentiation of stem cells, (3) responding osteogenic cells that form new bone, and (4) a sufficient vascular supply [6]. The contemporary treatment landscape encompasses multiple strategies that attempt to restore local biology by providing one or more of these elements [7]. Small defects (<1 cm) with preserved soft tissues may heal with conservative measures but can be managed with autologous bone graft, an allograft such as demineralized bone matrix, or growth factor delivery (i.e., recombinant bone morphogenetic protein (BMP)). Larger defects or those with a compromised soft tissue envelope (i.e., injury to the periosteum, vascular supply, and/or adjacent musculature) present a more complex reconstructive challenge. Sophisticated surgical techniques have been developed including the induced membrane technique and distraction osteogenesis; both techniques necessitate multiple surgical procedures and prolonged treatment regimens [2].

At present, there is no gold standard for the treatment of critical-sized bone defects. An ideal therapy would provide osteoconductive and osteoinductive elements while eliminating donor site morbidity and the need for multiple procedures or prolonged treatment regimens. Regional gene therapy has emerged as a potential tissue engineering solution that addresses current limitations [8]. In cell-based gene therapy approaches, osteoprogenitor cells can be genetically modified to express an osteoinductive protein, loaded onto an osteoconductive scaffold, and then implanted in the site of bone loss to exert a sustained therapeutic effect.

The purpose of this review is to discuss the current treatment options for the management of critical-sized bone defects along with their associated benefits and limitations. Furthermore, we aim to highlight regional gene therapy as a promising treatment option for the management of bone loss. We will discuss gene delivery methods, vector choice, candidate transgenes, and suitable cell types, as well as future directions for application in clinical practice.

## 2. Current Strategies for Segmental Bone Loss

### 2.1. Bone Grafts

#### 2.1.1. Autologous Bone Graft

Autologous bone grafting remains the reference standard for the management of fracture nonunion and segmental bone defects, with approximately 500,000 procedures performed annually in the United States [9,10]. Autologous bone grafting involves harvesting osseous tissue from one anatomic site and transplanting it to the site of the bone defect. Autografts can be harvested from multiple sites including the iliac crest, intramedullary canal (via reamer–irrigator–aspirator technique), medial femoral condyle, proximal tibia, distal radius, and calcaneus [6,9,10,11]. Depending on the harvest site, autografts may be either cortical (i.e., providing some structural support), cancellous, or both.

Autografts offer multiple advantages. The grafts contain complete tissue and are osteoconductive, osteoinductive, and osteogenic. In addition, they are fully biocompatible and avoid the risk of disease transmission [6]. Autografts can be harvested from multiple anatomic sites as mentioned above using relatively straightforward techniques [11].

However, there is a finite supply of autografts, which may pose a challenge in treating large defects or when multiple grafting procedures are needed. Harvesting autografts also requires a second surgical site, increasing operative time and blood loss, and is associated with well-known complications such as harvest site pain [12]. Moreover, the quality of the autograft may be compromised in elderly patients or those with medical comorbidities [13]. These limitations have driven the ongoing search for alternative bone graft substitutes.

#### 2.1.2. Allogeneic Bone Graft

Allogeneic bone grafts, or allografts, are derived from cadaveric donor tissue for transplantation into recipients. The primary advantages of allografts are that they are immediately available, come in a range of sizes, and avoid donor site morbidity. Allografts are available in various forms including cortical (i.e., structural) or cancellous graft, or demineralized bone matrix (DBM), with each suited to particular clinical applications [14].

Processing methods influence allograft properties and clinical utility. These methods include fresh, fresh-frozen, freeze-dried, demineralized, and gamma-irradiated allografts. Fresh-frozen cortical allografts provide structural integrity, retain some osteoinductive and osteoconductive properties, and can be size-matched to the defect, acting as an intercalary graft. Still, their use for the management of critical sized defects is associated with up to a 50% complication rate [15]. Moreover, these grafts contain immunogenic donor tissue and carry the risk of disease transmission including HIV, Hepatitis B or C, and bacterial infection [6]. Alternative processing techniques reduce immunogenicity and disease transmission risk but do so at the expense of structural integrity, as well as the biologic activity of the grafts [16]. Allografts alone have been shown to be inferior to autografts in the management of long-bone fracture nonunion, with longer time to union and higher re-operation rates [17].

DBM represents a form of allograft in which the mineral component of bone is extracted with acid, leaving the native proteinaceous components including type I collagen and osteoinductive growth factors such as BMPs, transforming growth factor β (TGF-β), and fibroblast growth factor (FGF) [18]. DBMs are predominately osteoconductive, and there is significant variability in biologic activity between products [19,20,21]. No high-level evidence supports the use of DBM alone for nonunions or critical-sized defects, and in clinical practice DBM is most commonly used as an adjunct to expand the volume of autograft [22,23].

Newer products such as Osteocel Plus (NuVasive Inc., San Diego, CA, USA), Trinity Evolution/ELITE (OrthoFix Medical Inc., Lewisville, TX, USA), map3 (LifeHealthcare, Randburg, South Africa), and ViviGen (Depuy Synthes, Raynham, MA, USA) combine allograft products with allogeneic osteoprogenitor cells to hypothetically enhance the osteoinductive and osteogenic properties. While small cohort studies in humans are promising, comparative data on efficacy are needed [24,25].

#### 2.1.3. Synthetic Bone Graft Substitutes

Synthetic bone graft substitutes are calcium-based ceramics such as calcium sulfate, calcium phosphate, tricalcium phosphate, and hydroxyapatite. The grafts are available in diverse formations including pellets, powders, putty, and solid blocks, providing a versatile osteoconductive scaffold. The advantages of synthetic grafts are that they avoid the risks and costs associated with autograft harvest, as well as the disease transmission risk of allografts; they are also available in an essentially unlimited quantity. However, they lack osteoinductive or osteogenic properties and, thus, are rarely used alone for the treatment of nonunion or segmental bone defects. A systematic review found that only 37% of bone graft substitutes available in the United Kingdom had any clinical data supporting their use, and only four of the more than fifty products evaluated had Level I evidence [26]. In current clinical practice, synthetic grafts are used as bone graft extenders, for backfilling small periarticular voids, or in the setting of infection when autografts or allografts could provide a nidus for colonization [27,28,29].

### 2.2. Cell-Based Therapies

Cell-based therapies for the management of nonunions and critical-sized defects include bone marrow aspirate injections and platelet-derived injections. These modalities are used as adjuncts to bone grafting procedures and are not used in isolation. Advantages common to both techniques include the use of autologous tissue and growth factors, as well as the limited morbidity associated with percutaneous harvest and delivery. Potential disadvantages inherent to cell-based therapies include quality variability based on patient demographic factors and harvest site, although conflicting reports on these topics have been published [13,30,31].

Bone marrow aspirate, which is generally harvested from the iliac crest, can be concentrated (BMAC) and is osteogenic, osteoinductive, and osteoconductive [32,33]. A systematic review of long-bone healing in preclinical models found that 100% of studies reported a significant increase in radiographic bone healing in the BMAC groups compared to controls; approximately 80% of studies reported improved biomechanical strength as well [34]. Clinical studies have produced more variable results, although BMAC is generally beneficial, but direct comparisons between studies are limited by differences in cell processing methods and study designs [35,36,37]. A major limitation of present-day techniques is that the concentration of osteoprogenitor cells contained within BMAC is unknown at the time of use.

Platelet-rich plasma (PRP) formulations contain numerous growth factors including platelet-derived growth factor (PDGF), TGF-β, insulin-like growth factor 1 (IGF-1), and vascular endothelial growth factor (VEGF); however, they do not contain BMPs. Their use in the treatment of nonunions and segmental defects is not currently supported by high-level evidence, but uncontrolled case series suggest potential efficacy [38]. In a randomized trial of 120 patients with long-bone nonunions, PRP was inferior to recombinant human BMP-7 (rhBMP-7) with regard to union rate and time to healing [39]. In difficult bone repair scenarios, PRP may lack a sufficient osteoinductive stimulus [40].

### 2.3. FDA-Approved Growth Factors for Bone Repair

Growth factors approved for clinical use include recombinant human BMP-2 and 7 (rhBMP-2 and 7) and recombinant platelet-derived growth factor (rhPDGF-BB) [41]. rhBMP-2 is the most well-studied growth factor for bone repair. BMP-2 is a potent osteoinductive stimulus that has an important role in osteogenesis and is constitutively expressed across the early phases of fracture healing [42,43]. rhBMP-2 is FDA-approved for use in acute open tibia fracture, anterior lumbar interbody fusion, and maxillofacial augmentation [44,45,46]. Its use for segmental bone defects remains off-label. While a robust preclinical body of literature exists for the management of critical sized defects with rhBMP-2, clinical evidence remains sparse. In the only randomized clinical trial to date, rhBMP-2 combined with an allograft demonstrated similar healing rates to autografts in the management of tibial diaphyseal defects (average defect size 4 cm) [47]. More widespread adoption of rhBMP-2 has been limited by a number of potentially serious side effects including soft tissue swelling, bone resorption, and heterotopic bone formation [48]. It is hypothesized that these side effects are related to (1) high doses of BMP-2 required to induce a sufficient osteoinductive response in humans, and (2) the rapid diffusion of the protein away from the collagen carrier, limiting the duration of targeted biological activity. Thus, the development of more efficient protein delivery systems as seen with gene therapy may allow for a dose reduction or a more sustained release that can enhance the efficacy of rhBMP-2 mediated therapies.

PDGF is active in the early phases of bone healing and is responsible for recruitment of MSCs and osteoblasts [49,50]. rhPDGF-BB is approved by the FDA for ankle and hindfoot fusions, and multiple clinical trials for these indications have shown that rhPDGF-BB combined with an osteoconductive calcium–ceramic scaffold achieves a similar fusion rate to an autograft [51,52]. However, its relatively modest osteoinductive potential may limit its utility for critical-sized defects, though further study is warranted [53,54].

### 2.4. Surgical Techniques for Large Defects

Autologous bone grafting is typically indicated for segmental defects up to 5 cm. For defects larger than 5 cm, additional surgical techniques have been described that include vascularized bone grafts, the induced membrane (Masquelet) technique, and distraction osteogenesis [2].

#### 2.4.1. Vascularized Bone Grafts

**Figure 1:**
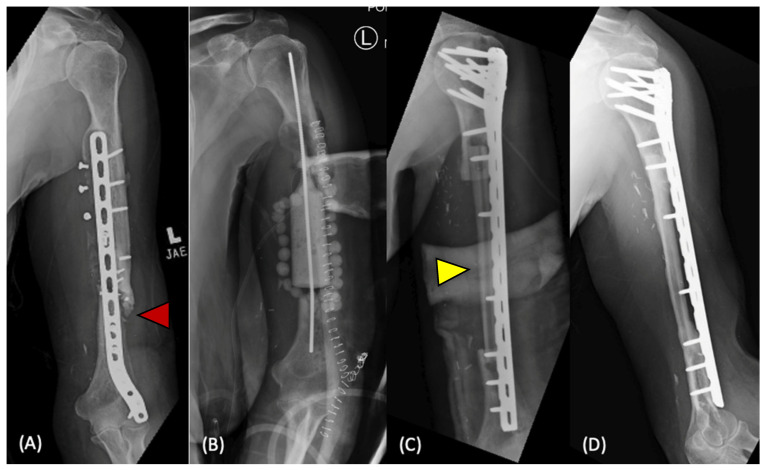
Management of an infected humeral shaft non-union (red arrow) (**A**). Initial management with hardware removal, debridement, placement of a temporary antibiotic cement spacer, and antibiotic beads (**B**). After treatment of the infection, the patient underwent vascularized free fibula transfer (yellow arrow) and revision open reduction internal fixation (**C**). Graft showed satisfactory incorporation one year after the procedure (**D**). Reproduced with permission from Mayfield et al. [61].

Vascularized bone grafting involves transferring an intact graft with its associated vascular supply, providing living bone with mechanical stability to the defect site (Figure 1). The procedures are technically demanding and require microsurgical expertise. Multiple graft options from upper and lower extremity sites are available, but the fibula is considered ideal for long-bone defects due to its shape, strength, and potential for hypertrophy [55,56]. Preservation of the graft’s native blood supply maintains osteogenic potential and promotes healing, which in turn reduces graft resorption and risk of mechanical failure compared to non-vascularized grafts [6]. Clinical studies have reported union rates from 77% to 100% in case series for indications including trauma, tumor, and infection [57,58,59,60]. Complications of vascularized bone grafting include loss of vascular supply, mechanical failure, and donor site morbidity; they also require a prolonged duration of partial weight bearing to permit adequate graft hypertrophy [56].

#### 2.4.2. Induced Membrane (Masquelet) Technique

The induced membrane technique, developed by Masquelet and Begue, is a two-stage procedure [62]. First, a polymethyl methacrylate cement spacer is placed in the bone defect for 4 to 8 weeks. During this time, the spacer induces the formation of a vascularized membrane rich in growth factors (BMPs, VEGF, TGF-β1, interleukin-6 (IL-6)) and osteoprogenitor cells [63,64]. A second procedure is then performed to incise the membrane, remove the cement spacer, and fill the void with bone graft. Clinically, the induced membrane technique has demonstrated reliable healing, with a recent meta-analysis of more than 1300 cases reporting an 82% union rate at an average of 6.6 months. Advantages of this technique include the use of a temporary cement spacer to preserve length and prevent interposition of soft tissues, as well as the creation of a vascularized, osteogenic space in what was previously a biologically unfavorable environment. Disadvantages of the technique include the need for multiple procedures, concomitant internal or external fixation, a long reconstructive period, and the potentially large volume of bone graft required [65].

#### 2.4.3. Distraction Osteogenesis

Distraction osteogenesis (i.e., bone transport), pioneered by Illizarov, involves the creation of new bone at a surgical osteotomy site that is then transported along the axis of the limb segment by the controlled application of mechanical strain [66]. In current practice, strain is applied via modern spatial external fixator frames or motorized intramedullary transport nails. Distraction osteogenesis induces features of both endochondral and intramembranous bone healing. After the osteotomy is made, there is a 5–10-day latent phase. Lengthening occurs during the distraction phase at a rate of 1 mm per day. The consolidation phase occurs once the transported segment spans the defect until union is achieved [67,68]. The advantages of distraction osteogenesis include the potential to bear weight during treatment, as well as the lack of donor site morbidity. Disadvantages include prolonged treatment time (average 6–12 months), high rates of pin-tract infections if external fixation is used, and a 5% rate of re-fracture [69].

## 3. Bone Tissue Engineering: Regional Gene Therapy

The currently available clinical interventions discussed in the prior section have all been shown to improve the repair of bone, but with notable limitations. Particularly in the setting of large-bone defects, current treatments fail to provide a consistently satisfactory solution. None possess all of the characteristics of an ideal bone substitute, as summarized by Amini et al.—high osteoinductive and angiogenic potentials, biological safety, low patient morbidity, no size restrictions, ready access to surgeons, long shelf life, and reasonable cost [70].

Bone tissue engineering (BTE) is an interdisciplinary field that has been borne out of and unmet and growing clinical need for bone graft, particularly in the settings of critical-sized defects and biologically compromised states (e.g., tumor, infection, and major trauma). BTE aims to develop strategies for restoring normal bone by combining biomaterials, cells, and growth factor delivery, eliminating shortcomings of current treatments. There are four critical elements in a traditional BTE model: (1) a biocompatible, osteoconductive scaffold, (2) osteogenic cells, (3) osteoinductive growth factors, and (4) adequate vascularization to support growth and remove waste products [71].

Regional gene therapy is a promising BTE strategy. In regional gene therapy approaches, nucleic acids or genetically modified progenitor cells are loaded onto an osteoconductive scaffold and placed into a bone defect to exert a sustained therapeutic effect, providing the necessary components for bone repair. We will provide an overview of this technology, with a particular emphasis on gene delivery methods, candidate transgenes, cell types, and preclinical results.

### 3.1. In Vivo Gene Delivery

**Figure 2:**
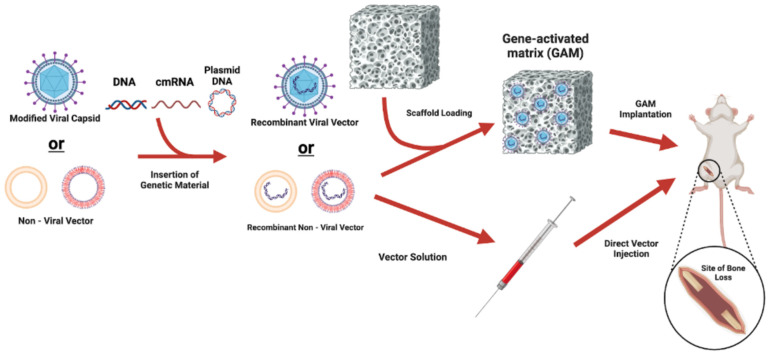
In vivo gene therapy for bone regeneration. Viral or non-viral vectors can be used to carry the genetic material of interest, such as DNA, circular mRNA (cmRNA), or plasmid DNA that encodes for an osteoinductive protein. Vectors can be combined with scaffold materials to create a gene-activated matrix that can be locally implanted with a surgical procedure. Alternatively, the vector with appropriate genetic material can be directly injected into the target tissue with or without a scaffold. Various chemical and physical processes have been developed, such as the use of liposomes or sonoporation, to improve gene transfer to host cells in vivo. (Figure created using BioRender.com).

Regardless of vector choice (i.e., viral or non-viral), there are two general strategies for gene delivery: in vivo and ex vivo. For in vivo gene delivery, the vector is introduced into the host at the site of the bone defect via direct injection or implanted as part of a matrix (Figure 2). This gene-activated matrix (GAM) typically refers to the combination of a non-viral vector and an osteoconductive scaffold with properties that allow for a more sustained release of genetic material [72].

In vivo strategies are relatively straightforward because the vector is delivered directly into the anatomic site. The major limitation is the reliance on a healthy population of responding cells, which are often absent in clinical cases of massive bone loss. Additional limitations include non-specific gene transfer and low gene transfer efficiency. If direct injection is used, an osteoconductive scaffold would not be implanted as part of the procedure, likely narrowing its applicability to small defects that may already be adequately treated with current methods. Table 1 summarizes the results of several preclinical studies utilizing in vivo gene delivery strategies.

**Table 1:** Selected preclinical studies using in vivo gene delivery for the treatment of critical-sized bone defects.

Author	Vector	Gene	Delivery Method	Animal Model	Findings
Fang et al. [73]	pDNA	PTH 1-34 BMP-4	GAM	Rat 5 mm Femoral Defect	Defect healing by 9 weeks in single plasmid (PTH 1-34 or BMP-4)-treated animals. More rapid healing and higher-quality bone formed in animals treated with both plasmids.
Bonadio et al. [74]	pDNA	PTH 1-34	GAM	Canine 8 mm Tibial Defect	PTH 1-34 expression detectable for 6 weeks in vivo. There was a dose-dependent effect on in vivo bone healing.
Baltzer et al. [75]	AV	BMP-2 TGF-β	Direct Injection	Rabbit 13 mm Femoral Defect	Radiographic healing by 12 weeks in 7/7 of treated animals. Superior biomechanical properties compared to control animals.
Geiger et al. [76]	pDNA	*VEGF*	GAM	Rabbit 15 mm Radial Defect	Most animals treated with VEGF exhibited partial or complete bone healing by 12 weeks, compared to none in controls. VEGF groups had 2–3 times the number of blood vessels by 6 weeks compared to controls.
Egermann et al. [77]	AV	*BMP-2*	Direct Injection	Sheep 20 mm × 5 mm Iliac Crest Defect; Sheep 3 mm Tibial Defect	Significantly reduced bone formation in animals treated with AV-BMP-2 injection compared to controls. Treated animals developed antibodies against the vector and human transgene.
Betz et al. [78]	AV	*BMP-2*	Direct Injection	Rat 5 mm Femoral Defect	A 100% healing rate in animals treated with high-dose AV-BMP-2 compared to 25% in the low-dose group. The high-dose group had higher bone volumes and bone mineral density, suggesting more robust bone formation and more rapid maturation.
Elangovan et al. [72]	pDNA	PDGF-B	GAM	Rat 5 mm × 2 mm Calvarial Defect	Complexes of polyethyleniminie-pPNA encoding for PDGF-B delivered on a collagen sponge resulted in up to 55-fold-greater new bone volume in animals relative to untreated controls.
Bez et al. [79]	pDNA	BMP-6	sonoporation	Mini-pig 1 cm tibial defect	Injection of pDNA microbubbles followed by delayed transcutaneous ultrasound (US)-mediated transfection produced union in 6/6 treated animals. Biomechanical properties of the US-treated animals were comparable to autograft.
Zhang et al. [80]	cmRNA	BMP-2	GAM	Rat 5 mm Femoral Defect	cmRNA encoding for BMP-2 was suspended in lipoplexes and delivered on a collagen sponge into defects. Bone formation was observed, but no defects had united by 8 weeks.

pDNA, plasmid DNA; cmRNA, chemically modified RNA; AV, adenovirus; PTH 1-34, teriparatide; BMP, bone morphogenetic protein; TGF-*β*, transforming growth factor beta; VEGF, vascular endothelial growth factor; PDGF-B, platelet-derived growth factor beta; GAM, gene-activated matrix.

### 3.2. Ex Vivo Gene Delivery

**Figure 3:**
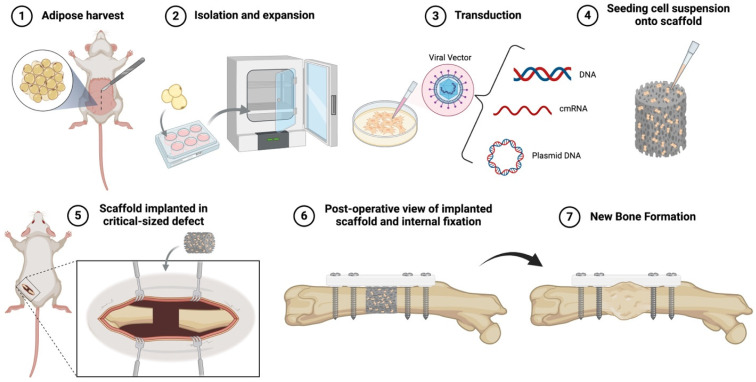
Ex vivo gene therapy with a viral vector for the management of critical-sized bone defects. Adipose-Derived Mesenchymal Stem Cells (ADSCs) are harvested and cultured from a donor rat. The ADSCs are then genetically modified using a viral vector to overexpress an osteoinductive protein (e.g., BMP-2). The modified ADSCs are then seeded onto a scaffold and implanted into the bone defect site. The genetically enhanced cells promote osteogenesis, which can be evaluated through radiographic, histological, and biomechanical analysis. (Figure created with BioRender.com).

In ex vivo gene delivery, vectors are used to genetically modify target cells outside the body, which are then seeded onto an osteoconductive scaffold and implanted at the site of bone loss (Figure 3). Typically, ex vivo methods involve the use of tissue culture expansion and genetic manipulation prior to use. The process may take hours to weeks depending on the cell type and the number of cells needed. Mesenchymal stem cells are typically used for ex vivo gene therapy, which may be autologous (i.e., harvested from the host) or allogeneic (i.e., harvested from a donor), as will be discussed in Section 5.4.

There are multiple advantages to using an ex vivo strategy: (1) target cell selection and expansion, (2) high transduction efficiency, (3) implantation of a healthy population of osteoprogenitor cells, and (4) potential for further stem cell expansion in vivo. If viral vectors are used, ex vivo approaches also eliminate the immunologic risks of systemic vector administration, although some degree of immune response could occur due to viral proteins present in transduced cells. Drawbacks of ex vivo approaches are the time and cost associated with ex vivo cell expansion and preparation. A selection of preclinical results using ex vivo gene delivery strategies for bone repair are summarized in Table 2.

**Table 2:** Selected preclinical studies using ex vivo gene delivery for the treatment of critical-sized bone defects.

Author	Species	MSC Cell Source	Vector	Gene	Scaffold	Animal Model	Results
Lieberman et al. [81]	Murine	Bone Marrow	AV	BMP-2	DBM	Nude Rat 8 mm Femoral Defect	Radiographic healing by 8 weeks in 3/3 animals treated with AV-BMP-2
Lieberman et al. [82]	Murine	Bone Marrow	AV	BMP-2	DBM	Rat 8 mm Femoral Defect	Radiographic healing by 8 weeks in 22/24 of treated animals. AV-BMP-2 group displaced thicker trabeculae compared to rhBMP-2-treated controls.
Tsuchida et al. [83]	Murine	(Allogeneic) Bone Marrow	AV	BMP-2	Collagen	Rat 6 mm Femoral Defect + Immunosuppression	Radiographic healing by 8 weeks in 8/8 of allogeneic + systemic tacrolimus-treated animals. Results were comparable to animals receiving transduced syngeneic cells.
Peterson et al. [84]	**Human**	Adipose	AV	BMP-2	Collagen–ceramic	Nude Rat 6 mm Femoral Defect	Radiographic healing by 8 weeks in 11/12 treated animals.
Virk et al. [85]	Rat	Bone Marrow	LV, AV	BMP-2	Collagen–ceramic	Lewis Rat 8 mm Femoral Defect	Radiographic healing by 8 weeks in 10/10 of LV-BMP-2-treated animals vs. 7/10 in AV-BMP2-treated animals. Superior biomechanical properties in LV-BMP-treated animals.
Hao et al. [86]	Rabbit	Adipose	AV	BMP-2	Novel biocomposite	White Rabbit 5 mm Radial Defect	Superior radiographic healing by 6 weeks in treated animals compared to controls. Complete healing and scaffold resorption by 12 weeks.
Virk et al. [87]	Murine	Bone Marrow	LV	BMP-2	Collagen–ceramic	Lewis Rat 8 mm Femoral Defect	Radiographic healing by 8 weeks in 13/13 “same day” treated animals. Earlier healing, greater bone volume, and superior biomechanical properties in “same day” vs. traditional 2-step ex vivo approach.
Qing et al. [88]	Murine	Adipose	LV	BMP-2 BMP-7	Calcium ceramic	Rat 2 mm Femoral Defect	Faster and more efficient healing noted at 6 weeks in BMP2 + BMP7 co-transfected treated animals.
De La Vega et al. [89]	Murine	(Allogeneic) Skeletal Muscle	AV	*BMP-2*	Muscle Disc	Rat 5 mm Femoral Defect	Enhanced bone formation by 8 weeks in AV-BMP-2 + immunosuppressed animals.
Bougioukli et al. [90]	**Human**	Bone Marrow	LV	*BMP-2*	Collagen–ceramic	Nude Rat 6 mm Femoral Defect	Radiographic healing by 8 weeks in 12/14 of traditional “two step” BMP-2-treated animals vs. 7/14 of “same day” treated animals.
Vakhshori et al. [91]	**Human**	Adipose	LV	*BMP-2*	Calcium ceramic	Athymic Nude Rat 6 mm Femoral Defect	Radiographic healing by 12 weeks in 13/14 treated animals. Comparable biomechanical qualities to positive control animals.
Kang et al. [92]	Rat	Bone marrow	LV	*BMP-2*	3D-Printed Hyperelastic bone	Lewis Rat 6 mm Femoral Defect	Radiographic healing by 12 weeks in 12/14 treated animals. Comparable biomechanical qualities to animals treated with rhBMP-2.

AV, adenovirus; LV, lentivirus; RV, retrovirus; AAV, adeno-associated virus; DBM, demineralized bone matrix, SCID, Severe Combined Immunodeficiency; nHA/RHLC/PLA, nano-hydroxyapatite/recombinant human-like collagen/poly(lactic acid); BMP, bone morphogenic protein; VEGF, vascular endothelial growth factor.

Due to the inherent time and resource expenditure associated with cell culture, expedited ex vivo methods have also been developed. With expedited methods, autologous tissue such as bone marrow, skeletal muscle, or fat can be harvested, genetically modified ex vivo, and then re-implanted with the goal of accomplishing this during the same operation. Both “next-day” and “same-day” strategies have been described [87,93,94,95]. Betz et al. described a “next-day” approach in which skeletal muscle was isolated from donor rats, transduced with an AV vector to express BMP-2, and re-implanted the next day into a segmental femoral defect in a syngeneic rat [93]. In total, 100% of the defects healed, and the newly formed bone was histologically and biomechanically comparable to a control group treated with autologous bone graft. Our group observed similar results using a “same-day” approach in which rat bone marrow cells were isolated, transduced to express BMP-2, and re-implanted into a rat segmental femoral defect, all within 3 h [87]. These results suggest a possible path towards more cost-effective clinical translation of autologous ex vivo gene therapy applications. However, it is important to recognize that expedited approaches implant far fewer transduced cells than traditional approaches, which may limit efficacy in cases of massive bone loss.

### 3.3. Viral Vectors

Viral-mediated gene transfer is termed transduction. Viral vectors utilize the ability of viruses to efficiently transfer their genetic material into human cells. Viral vectors are genetically modified to remove sequences involved in pathogenicity, providing space for desired transgenes and their regulatory elements. Viral vectors can be categorized by features including tropism, genome integration, immunogenicity, and genetic carrying capacity. Numerous viruses have been investigated as possible gene therapy vectors, but the most commonly utilized are adenovirus (AV), adeno-associated virus (AAV), and lentivirus (LV) [96]. A summary of vector properties is listed in Table 3.

AV vectors were among the first adopted for clinical use and remain the most well studied, accounting for approximately 50% of all current gene therapy trials [96,97]. They have been primarily used in anticancer therapy and in the development of novel vaccines, including for SARS-CoV-2. Advantages include their broad tropism for different cell types, high transduction efficiency, lack of genome integration, which eliminates the risk of insertional mutagenesis, and large packaging capacity. The main disadvantages of AV vectors are the strong host immune response to the viral proteins and the limited duration of transgene protein expression [98,99]. This could limit the efficacy of regional gene therapy in clinical scenarios where large bone defects are present and an extended duration of osteoinductive gene expression is needed.

AAV vectors are an increasingly popular choice across a spectrum of gene therapy applications, now used in over 100 clinical trials worldwide for a variety of monogenic diseases including hemophilia and spinal muscular atrophy [100]. Compared to AV vectors, AAV vectors have reduced immunogenicity and vector toxicity, as well as increased duration of transgene expression [96,101]. Moreover, they are considered relatively safe, as AAV vectors are non-integrating and are not linked to any known causes of human disease. Unfortunately, production of AAV vectors remains complex and costly, which is a major hurdle for clinical use. The packaging capacity is also limited to ~5 kb, which could be a drawback for certain applications requiring large transgenes or expression constructs [102]. However, most bone repair applications focus on the delivery of osteoinductive factors (e.g., BMP-2), where the complementary DNA (cDNA) is relatively small and should fit within this limit. Another limitation of AAV vectors was reported by Bougioukli et al., who noted that AAV serotypes 2 and 6 had a limited ability to transduce human MSCs [103]. Future studies on AAV vectors for bone tissue engineering applications may need to evaluate other serotypes (12 have been described) or modify the transduction conditions to improve gene transfer efficiency.

LV vectors, a type of retrovirus, are derived from the human immunodeficiency virus 1 (HIV-1). The primary advantage of LV vectors is the stable incorporation of viral DNA into the host genome, resulting in long-term transgene expression. However, this property also poses a unique safety concern: the risk of insertional mutagenesis [104,105]. Additionally, given its derivation from HIV-1, there is the theoretical risk of generating a replication competent provirus. Successive generations of LV vectors have focused on improving safety, with the removal of non-essential viral proteins and the separation of the genetic material into three plasmids. Contemporary third-generation, self-inactivating LV vectors have helped to mitigate the risk of virus reconstitution and insertional mutagenesis [106,107,108]. Still, it is critical to underscore the importance of safety, particularly in gene therapy applications for non-lethal conditions such as bone loss [109,110]. LV vectors have now been used clinically for a number of conditions including chimeric antigen receptor T-cell therapy, beta thalassemia, and sickle cell disease [111].

Prior studies from our laboratory have evaluated the safety of third-generation LV vectors for regional gene therapy applications for bone repair. We have shown in two large biodistribution studies that mesenchymal stem cells transduced ex vivo and implanted into critical-sized femoral defects in rats show a non-specific pattern of biodistribution, no histologic abnormalities across multiple organs, and no tumorigenesis out to 12 weeks post-implantation [112,113]. Moreover, integration site analysis revealed polyclonal transduction and no expanded clones with integration near cancer-associated genes.

To further increase the theoretical safety of LV vectors, the use of “suicide” gene approaches or inducible expression systems have been developed to selectively eliminate vector-carrying cells [114,115]. These systems are designed so that the expression of apoptotic pathways or the production of toxic metabolites can be activated by the administration of an activating substance, typically after the desired therapeutic effect is achieved. In a study by Alaee et al., cells were transduced with a bicistronic vector encoding BMP-2 and a modified viral thymidine kinase (Δtk) [115]. Administration of ganciclovir led to the formation of toxic metabolites in transduced cells and, when used in vivo, resulted in an absence of bone formation in a mouse critical-sized femoral defect model. A major limitation of these “suicide” strategies is a decrease in osteoinductive protein production due to the presence of two transgenes.

### 3.4. Viral Vectors—Preclinical Results for Bone Repair

Viral vectors have shown efficacy across multiple preclinical models for bone loss including calvarial defects, posterior spine fusion, and long-bone segmental defects (Table 1 and Table 2 provide a selected list of studies) [8,75,81,116,117]. However, it should be noted that the majority of this research has been performed in rodent models. Rodent species are often syngeneic and generally exhibit rapid bone healing, providing a reproducible, time-efficient, and cost-effective model for studying bone regenerative therapies [118,119]. Studies in clinically relevant large animal models are more limited, and further research is clearly needed to convincingly demonstrate efficacy in large animal models prior to clinical translation [77,79,120].

In a pioneering study, Lieberman et al. used a first-generation AV vector to transduce autologous rat bone marrow cells, which were then loaded onto a DBM carrier and implanted in an 8 mm critical-sized femoral defect in a Lewis rat [82]. More than 90% of the defects (22 of 24) had completely healed by 8 weeks, and the healed femora had biomechanical properties comparable to untreated controls. Since that time, other groups have also successfully used AV vectors with both ex vivo and in vivo gene therapy approaches for bone repair [75,121].

Early studies with AV vectors for bone repair also highlighted the provoked immune response as a potential limitation to efficacy. Alden et al. demonstrated that injection of a BMP-2 AV vector produced robust ectopic bone in an immunodeficient rat, whereas immunocompetent rats had minimal bone formation due to a massive inflammatory response [122]. Concordant findings have been reported in numerous other studies [77,95,117,123]. Furthermore, improved bone healing is seen when immunocompetent animals treated with an AV vector are simultaneously immunosuppressed with systemic medications [124,125,126,127].

Despite the potential of AV gene therapy, there was concern that short-term transgene expression and immunogenicity of the vector would result in an attenuated or inconsistent biological response in humans, which could lead to treatment failure in cases of massive bone loss or biologically stringent environments (i.e., poor soft tissue coverage and vascularization). In general, AV vector use produces approximately 3 weeks of transgene expression in vivo in rats [85,128]. Given differences in immunity between humans and preclinical models, it is possible that the duration of transgene expression would be even shorter in a clinical setting [129]. Our group has since investigated lentiviral vectors for prolonged transgene expression. The initial studies demonstrated excellent efficacy. Rat bone marrow cells transduced with a LV vector to express BMP-2 and implanted in a hindlimb muscle pouch produced robust ectopic bone [130]. Subsequent studies in rat critical-sized defects and rat spine posterolateral fusion models resulted in 100% union rates [131,132]. When comparing AV and LV vectors, we have found that LV vectors produce a significantly longer duration (weeks vs. months) of transgene expression in vitro and in vivo, which results in greater bone volume and biomechanical strength in vivo [85,128,131]. While we have observed more robust healing responses utilizing LV vectors, other groups have purported benefits of relatively transient transgene expression in similar models using AV vectors [125,133].

### 3.5. Non-Viral Vectors

Non-viral-mediated transfer is termed transfection. Non-viral vectors are a potential alternative to viral vectors, circumventing concerns such as immunogenicity, insertional mutagenesis, and cost. The simplest example of a non-viral vector is the injection of naked plasmid DNA (pDNA), but the transfection efficiency of naked pDNA is quite low. Physical or chemical means are used to improve transfection efficiency. Examples of physical methods include electroporation, sonoporation, and magnetofection, which induce a temporary increase in cell membrane permeability. Examples of chemical methods include complexes of lipids, polymers, or nanoparticles that improve cellular uptake or endocytosis. Detailed reviews of non-viral vector methods have been recently published [134,135].

Historically, non-viral gene transfer is less efficient compared to viral transduction, limiting transgene expression. Thus, non-viral approaches for bone repair application are less common given the goal of robust expression of critical growth factors. Even so, multiple groups in the past two decades have used non-viral vectors in preclinical studies showing proof of concept and efficacy (selected studies are summarized in Table 1) [79,136,137]. Bez et al. demonstrated the potential of sonoporation-mediated BMP-6 delivery [79]. Using a Yucatán mini-pig model, a 1 cm tibial defect was created, and 14 days after the procedure, a microbubble solution of BMP-6 pDNA was percutaneously injected into the defect site. An external ultrasound pulse was then given over the defect site to trigger transfection of the local cells. All six (100%) of the treated pigs achieved complete radiographic healing within six weeks, and the tibiae were histologically and biomechanically comparable to autograft-treated animals.

## 4. Gene Candidates

**Figure 4:**
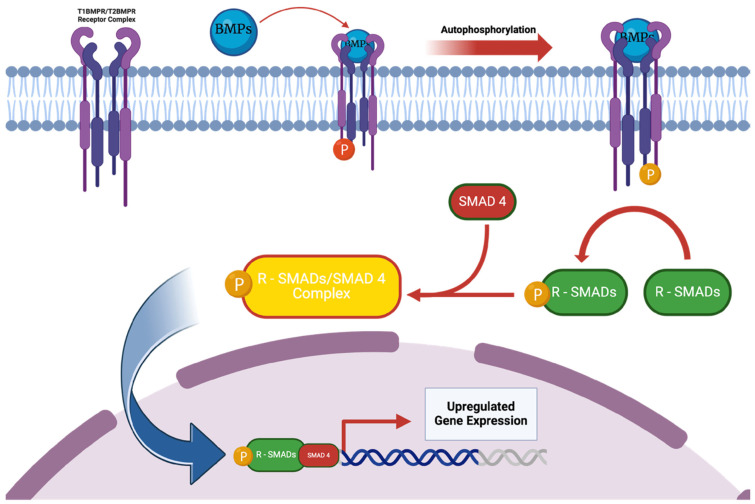
Canonical BMP signaling pathway. BMPs bind to a receptor complex made up of Type I and Type II BMP receptors (T1BMPR/T2BMPR) on the cell membrane. Upon BMP binding, the receptor undergoes autophosphorylation, activating the intracellular signal transduction process. Phosphorylated receptor-regulated SMADs (R-SMADs) then associate with SMAD4, forming an R-SMAD/SMAD4 complex. This complex translocates into the cell nucleus, where it binds to specific DNA sequences, upregulating expression of genes contributing to osteogenesis. (Figure created using BioRender.com).

An expanding list of genes has been studied in the context of regional gene therapy for bone repair [133,138]. Among them, BMP-2 remains the most promising, as it has a well-characterized signaling pathway (Figure 4), a critical role in multiple phases of bone healing, a potent osteoinductive effect, and an extensive body of literature on its effects in animals and in humans [48,139,140,141]. Moreover, BMP-2 is FDA-approved, as was discussed in Section 2.3, which would help to facilitate the clinical translation of a BMP-2 gene therapy application.

Numerous other genes involved in osteogenesis have been investigated including other BMPs, TGF-β, PDGF, FGF, insulin-like growth factor 1 (IGF-1), RUNX-2, and osterix, as well as genes involved in angiogenesis such as VEGF and cyclooxygenase-2 [8,133].

Combinatorial gene therapy approaches have also proven successful such as the use of multiple osteoinductive factors, or osteoinductive and angiogenic factors together, but it would be challenging to obtain FDA approval for this type of strategy [137,142,143,144]. In an alternative approach, Bougioukli et al. showed that gene therapy could be combined with systemic agents to improve bone healing [145]. They found that ex vivo gene transfer of BMP-2 combined with systemic osteoprotegerin (a receptor activator of nuclear factor kappa-Β ligand (RANKL) inhibitor that blocks differentiation and function of osteoclasts) produced better bone healing than BMP-2 alone in a mouse critical-sized bone defect.

Locally acting inhibitors have also been studied using small interfering RNA (siRNA), which creates temporary gene knockdowns. These approaches may help to mitigate homeostatic responses to therapeutic products. For example, BMP-2 causes upregulation of noggin expression, a BMP-2 inhibitor. Kowalczewski et al. showed that in cells treated with BMP-2, nonviral delivery of siRNA against noggin resulted in a near absence of noggin protein [146]. In another recent study, Mora-Raimundo et al. utilized a non-viral vector to deliver a combination of SOST siRNA, which promotes osteoblast differentiation and osteostatin, a osteogenic peptide [147]. The authors found that co-delivery of these biomolecules produced a synergistic effect on osteogenic gene expression in vivo in a mouse model. siRNA could be delivered to cells using viral or non-viral gene delivery methods, highlighting the exciting potential of these approaches to enhance therapeutic response [148].

Another emerging technology is CRISPR/Cas9, a powerful tool for gene editing that can be delivered to target cells using viral or non-viral gene delivery methods. This approach may allow for the upregulation and downregulation of multiple genes simultaneously. As it remains in its infancy for bone healing applications, it will not be discussed in detail in this review. Its application in the treatment of other musculoskeletal pathology have been reviewed elsewhere [149,150].

## 5. Stem Cells for Ex Vivo Regional Gene Therapy

Stem cells are promising vehicles for ex vivo regional gene therapy applications, offering the potential to deliver therapeutic transgenes while also serving as a source of osteoprogenitor cells that can contribute to the bone repair process. Therefore, the osteogenic potential of a stem cell source is an important consideration when determining its suitability for gene therapy applications. In addition to osteogenic potential, an ideal autologous stem cell source would have the following characteristics: easy to obtain with minimal or no patient morbidity, high yield and rapid expansion in cell culture, and permissive to gene transfer. Immunogenicity is also important to consider as it opens the possibility of allogeneic gene therapy approaches.

**Figure 5:**
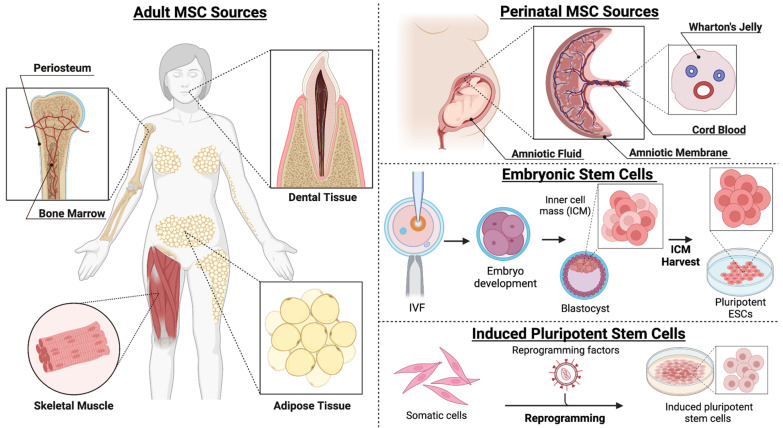
Stem cell sources for bone tissue engineering applications. Adult mesenchymal stem cells (MSCs) are found in various tissues but are most commonly isolated from adipose tissue, bone marrow, skeletal muscle, and dental tissues in bone tissue engineering applications. Perinatal MSCs can be isolated from birth associated tissues. Embryonic Stem Cells are derived from the inner cell mass of the blastocyst and are totipotent. Induced Pluripotent Stem Cells are adult cells that have been reprogrammed into an “embryonic cell-like state” and are pluripotent. (Figure created with BioRender.com).

Several types of stem cells, derived from various tissue sources, have been explored in the context of gene therapy for bone regeneration (Figure 5). However, prior investigations have mainly focused on mesenchymal stem/stromal cells (MSCs) due to their chondrogenic and osteogenic potential. Autologous cells are most frequently used in preclinical studies, although approaches with allogeneic cells have increasingly become a topic of interest.

It should be noted that the isolation, characterization, and application of stem cells for bone tissue engineering are also covered in reviews on cell-based therapeutics, but these approaches utilize non-genetically modified cells [151]. Given the breadth of the topic, the focus of our review will be on stem cell types and sources with preclinical data for gene therapy applications.

### 5.1. Embryonic Stem Cells (ESCs)

ESCs are pluripotent cells derived from the inner cell mass of blastocysts and can differentiate into any cell type, including osteogenic lineages [152]. ESCs have been shown to differentiate into osteogenic cells in vitro and promote bone formation in vivo [153,154]. However, the use of ESCs is associated with a number of technical and ethical concerns. Due to their pluripotency, they typically must be differentiated in vitro before being used in vivo. Obtaining a purified cell lineage is difficult compared to multipotent stem cells and carries with it the risk of unintended differentiation or teratoma formation in vivo, the latter of which would be unacceptable when treating a non-lethal condition like bone loss [155]. ESCs are also immunogenic, capable of eliciting host immune responses and potentially mitigating therapeutic benefit [156,157]. The process of obtaining ESCs is also fraught with ethical concerns because the embryo is destroyed in the process [158]. Comparative in vivo studies do not support the ESCs as a superior choice for bone tissue engineering applications; murine and human ESCs demonstrate equivocal or inferior in vivo bone formation compared to bone-marrow-derived MSCs (BMSCs) [159,160]. To our knowledge, no studies have evaluated ESCs in the context of gene therapy for bone repair.

In light of the above, adult and perinatal stem cell sources are preferable for ex vivo gene therapy applications for bone repair as they are considered safer and equally effective, while also avoiding the same ethical concerns of ESCs.

### 5.2. Induced Pluripotent Stem Cells (iPSCs)

iPSCs are derived from adult somatic cells that have been reprogrammed to a pluripotent state, enabling them to differentiate into any cell type. Yamanaka and colleagues first achieved this with mouse somatic cells in 2006; a year later, their group successfully reprogrammed human skin fibroblasts in the same way, which involved the retroviral transfer of four transcription factors: Oct3/4, Sox2, Klf4, and c-Myc [161,162]. Since then, various adult cell types have been utilized to generate iPSC-derived MSCs for bone tissue engineering applications [163].

The main advantages of iPSCs are that they overcome the immunologic and ethical concerns associated with ESCs by using autologous tissue. They can be harvested in a minimally invasive manner from almost any adult tissues, and reprogramming restores an embryonic proliferation rate and differentiation potential. However, tumorgenicity is still considered a risk with iPSCs, and there is additional time and cost associated with cell reprogramming and differentiation, all of which must be considered when looking ahead towards clinical translation [164,165]. iPSC-derived MSCs have demonstrated good osteogenic potential in vitro and in vivo, but few studies to date have evaluated their potential in gene therapy applications for bone repair [166,167,168,169].

### 5.3. Mesenchymal Stem Cells (MSCs)

MSCs are the most widely studied cell type for orthopedic gene therapy, owing to their presence in multiple tissues, rapid expansion in culture, capacity for genetic modification, and ability to differentiate into bone or cartilage tissues under the influence of an osteoinductive transgene such as BMP-2 [170,171]. MSCs also possess broad immunomodulatory properties, which may serve to enhance bone repair or permit allogeneic cell-based approaches [172,173,174]. First isolated from bone marrow, MSCs or MSC-like cells have now been isolated from numerous tissue sources. A summary of MSC sources and properties are provided in Table 4.

#### 5.3.1. MSC Heterogeneity

MSC heterogeneity is an important factor in MSC-based gene therapy, with variations arising from (1) different donors, (2) different tissue sources, and (3) intercellular differences within the same tissue source [175].

Donor differences, particularly the effect of aging, are important to consider. With aging, MSC content in bone marrow decreases, and the MSCs may exhibit reduced proliferation rates, osteogenic potential, and transgene expression [90,176,177,178]. This variability could potentially impact treatment response. In addition to the cells, the age of the recipient is also an important factor. Gao et al. found that in vivo bone formation was affected by a recipient rat’s age rather than the age of donor cells (BMP-2 transduced human muscle-derived MSCs) [179]. These findings underscore the potential impact of both cell and host biology on treatment response.

MSCs from different tissue sources also vary in their MSC content, proliferative capacity, immunomodulation, gene transfer efficiency, and differentiation capacity [175]. Even MSCs from the same tissue source in different anatomical locations (e.g., subcutaneous adipose vs. visceral adipose) also show considerable variation [180,181].

#### 5.3.2. Bone-Marrow-Derived Stem Cells (BMSCs)

BMSCs were first described in 1966 by Friedenstein, although the term “mesenchymal stem cells” was not coined until 1991 when Caplan further characterized this unique cell population with self-renewal capabilities and multipotent differentiation into adipocytic, chondrocytic, and osteocytic lineages [182,183]. To date, BMSCs are the most extensively characterized and well studied in the field of bone tissue engineering and gene therapy for bone repair. Furthermore, BMSCs are typically used as the comparator when evaluating alternative MSC sources [184,185,186].

BMSCs were the first stem cell to be successfully used as part of an ex vivo gene therapy approach for bone regeneration [81,82]. These studies demonstrated high transduction efficiency, sustained BMP-2 production, in vitro osteogenic potential, and, most importantly, the ability to heal a critical-sized bone defect. Efficacy was confirmed in similarly designed studies, and proof of concept with human BMSCs has also been demonstrated both in vitro and in vivo [8].

A limitation of BMSCs is that their harvest requires bone marrow aspiration, a separate surgical procedure. Another disadvantage of BMSCs is the low yield of MSCs obtained from aspiration. Compared to bone marrow, adipose tissue yields a nearly 500-fold greater increase in MSC content [187]. Furthermore, the growth and differentiation characteristics of BMSCs are affected by a patient’s age and by demographics such as sex [178,188,189]. Identifying a MSC source that is less affected by these factors would support the translation of an autologous cell-based gene therapy approach.

#### 5.3.3. Adipose-Derived Stem Cells (ADSCs)

Multipotent stem cells were first isolated from fat in 1964 by Rodbell, later termed adipose-derived stem cells (ADSCs) [190]. They have garnered significant interest for bone tissue engineering and ex vivo gene therapy applications because of their unique biological properties and ease of harvest in human patients [191]. Adipose tissue is obtainable in large quantities and with minimal morbidity using suction-assisted lipectomy (i.e., liposuction) or from normally discarded tissue in common surgical procedures such as the infrapatellar fat pad when performing total knee arthroplasty [192,193]. Compared to bone marrow, adipose tissue has a higher yield of MSCs, and these MSCs grow more quickly in cell culture [175,194]. Additionally, multiple studies have shown that ADSCs possess stronger immunomodulatory properties than BMSCs, but this potential declines with the age of the donor [195,196,197]. This suggests that ADSCs may be suitable for allogeneic approaches using cells from a young, healthy donor. Comparative studies have also evaluated osteogenic potential, with a recent review favoring BMSCs in vitro but finding comparable results in vivo [198]. Importantly, the osteogenic and chondrogenic potential of ADSCs appears less affected by donor age than in BMSCs [199,200]. Particularly relevant to gene therapy is that ADSC donor characteristics do not seem to affect transgene expression and osteogenic potential [176].

ADSCs have proven to be valuable vehicles for gene therapy applications for bone regeneration. Human processed lipoaspirate (containing ADSCs) transduced to overexpress BMP-2 underwent transition to an osteoblastic lineage at a comparable rate to cells treated with rhBMP-2. Further, this transition occurred more quickly, and the resultant bone formation was greater than in transduced BMSCs [201]. Subsequent in vivo studies demonstrated that BMP-2-transduced ADSCs produced abundant bone formation in vivo in multiple models (ectopic, segmental defect, and spine fusion) [84,201,202]. In a critical-sized defect model, Peterson et al. found that non-transduced ADSCs alone did not form bone, highlighting the importance of an osteoinductive stimulus to guide lineage differentiation in vivo. Importantly, it has been shown that transgenic BMP-2 is a more potent osteoinductive stimulus than rhBMP-2, which supports an integrated gene therapy approach compared to the combination of recombinant growth factors and non-transduced MSCs [203].

ADSCs are widely used in contemporary preclinical gene therapy approaches for bone repair [91,112,204,205,206]. In an important comparative study for gene therapy approaches, Bougioukli et al. demonstrated that human ADSCs have increased transduction efficiency with a LV vector, increased BMP-2 transgene expression, and increased osteogenic differentiation capacity compared to BMSCs, supporting ADCSs as an superior choice [204]. Transduced human ADSCs have also shown excellent therapeutic potential in vivo. In a critical-sized defect model using immunocompromised rats, Vakhshori et al. found that human ADSCs transduced with an LV vector to overexpress BMP-2 produced complete radiographic healing in 13 of 14 animals in 12 weeks; the healed femora had biomechanical properties comparable to animals treated with rhBMP-2 [91].

#### 5.3.4. Muscle-Derived Stem Cells (MDSCs)

Yaffe first isolated stem cells from skeletal muscle in 1968 [207]. In subsequent work, Qu-Petersen et al. identified a subpopulation of these stem cells with MSC-like characteristics, termed muscle-derived stem cells (MDSCs) [208]. MDSCs have since been isolated in humans and have been shown to have osteogenic potential in vitro and in vivo [208,209]. An advantage of MDSCs is that procurement can be accomplished with a muscle biopsy, an in-office procedure performed under local anesthesia. A challenge with MDSCs is the cell isolation process, which is time and resource intensive; it can take weeks to isolate a population of MDSCs, which must then be expanded to a workable quantity [210,211].

In vitro and in vivo studies have demonstrated that BMP-2 is sufficient to induce osteogenic differentiation of both murine and human MDSCs [212,213,214]. Genetic engineering of MDSCs also creates an effective therapy. In a study by Lee et al., human MDSCs were transduced to express BMP-2 and implanted in calvarial defects of immunodeficient mice, with all animals demonstrating complete healing by 8 weeks. The study also evaluated the fate of the implanted MDSCs, which revealed that the implanted cells had differentiated in vivo and incorporated into newly formed bone [214]. Additional gene therapy studies with MDSCs have demonstrated similarly promising results in vivo using various transgenes such as BMP-2, BMP-4, and combinations of genes [121,215,216]. Work by Wright et al. also showed that allogeneic MDSCs transduced with BMP-4 were able to heal critical-sized defects in rats despite the presence of an immune reaction [216]. A subsequent study found that implanted MDSCs partially suppressed host immune responses through the secretion of various trophic factors [217]. In contrast, allogeneic muscle “grafts” transduced with BMP-2, which were developed as part of an expedited ex vivo approach, healed only 33% of calvarial defects in immunocompetent rats, compared to 100% if autologous cells were used [95]. This finding is not surprising, considering that primary muscle tissue is more immunogenic than MDSCs, but again points to the central role of the immune system in effective therapeutic response [218].

#### 5.3.5. Dental-Derived Stem Cells

Dental-derived MSCs, first isolated from dental pulp tissues (DPSCs) by Gronthos et al. in 2000, have since been discovered in various dental tissues [219,220]. These MSCs are promising for maxillofacial bone regeneration due to the low morbidity of harvest (i.e., isolated from extracted wisdom teeth or other dental tissues discarded as medical waste), high proliferative capacity, and low immunogenicity, particularly as compared to BMSCs [221,222,223]. However, overall yield is a concern, potentially limiting broader application in scenarios requiring more cells, such as long bone segmental defects.

Dental-derived MSCs have demonstrated osteogenic potential in vitro and bone formation in vivo in preclinical models. MSCs derived from the periodontal ligament or exfoliated deciduous teeth seem to have the highest osteogenic potential in vitro, although multiple cell types have shown effectiveness for in vivo bone regeneration [224,225,226,227]. In vivo comparisons to BMSCs and ADSCs have produced mixed results, requiring further study in more stringent models [228,229,230]. Dental-derived MSCs have also been used in gene therapy applications, showing robust ectopic bone formation and segmental defect healing when genetically modified with either BMP-2 or BMP-7 [231,232,233].

#### 5.3.6. Other Adult MSC Sources

As adult MSCs can be isolated from nearly any tissue, there is an expanding list of MSC sources including the periosteum, skin, peripheral blood, and urine. However, these cell sources may be challenging to harvest, lack efficacy compared to the cell types described above, or presently lack robust data to support their role in gene therapy approaches for bone repair [151].

The periosteum is a source of skeletal stem cells and plays a critical role in the normal bone repair process [234]. However, its procurement in large quantities is not feasible given the associated morbidity. Few studies have used periosteal stem cells for gene therapy approaches [235]. Skin is a source of MSCs that can be obtained with biopsy and readily regenerates; gene therapy approaches using skin have been able to produce bone in vivo [236,237]. Peripheral blood also contains MSC-like cells with multipotent differentiation and can be harvested in a minimally invasive, low-cost procedure [238,239,240]. To this point, they have not been utilized in gene therapy approaches for bone healing but have shown osteogenic potential equal to BMSCs in other tissue engineering applications [241,242]. In the past decade, urine-derived MSCs have emerged as an alternative for bone tissue engineering applications, in part due to easy, non-invasive procurement. These cells have demonstrated osteogenic potential and can produce bone in vivo, although no gene therapy studies to date have utilized this cell source [151,243].

Peripheral blood- and urine-derived MSCs are particularly promising cell sources given their availability, ease of harvest, and demonstrated efficacy in the limited studies conducted to date. As more becomes known about these stem cells, they may become attractive options for gene therapy researchers.

#### 5.3.7. Perinatal Mesenchymal Stem Cells

Perinatal MSCs, first isolated from the umbilical cord by Covas (cord tissue) and Romanov (cord blood) in 2003, have since been isolated from other tissues including Wharton’s jelly, amniotic membrane, amniotic fluid, chorionic membrane, and decidua. The primary advantage of this stem cell source is that there is an abundance of perinatal tissues, which are typically considered medical waste, providing an inexhaustible source of cells without imparting morbidity on the mother or child. However, cell-based therapies with perinatal MSCs would be allogeneic, raising immunogenicity concerns. Perinatal MSCs, particularly umbilical cord and amniotic fluid/membrane, are promising stem cell sources for allogeneic gene therapy due to their uniquely low immunogenicity and favorable immunomodulatory properties, which may also influence the microenvironment in a way that promotes bone healing [244,245,246,247,248,249,250].

Among perinatal tissues, MSCs isolated from amniotic membrane and umbilical cord have the highest osteogenic potential [251,252]. Compared to other adult stem cell tissue sources, perinatal MSCs have higher in vitro osteogenic potential than BMSCs but lower than ADSCs; in vivo reports have been mixed [253,254,255]. Perinatal MSCs can be efficiently modified with viral and non-viral gene delivery methods and have been used in a variety of ex vivo gene therapy contexts, though few for bone tissue engineering [256,257,258,259,260]. Recently, Bougioukli et al. demonstrated >90% transduction efficiency of human umbilical-cord-derived MSCs using a lentiviral vector encoding the cDNA for BMP-2 [256]. Transgene expression of BMP-2 induced osteogenic differentiation in vitro and produced robust bone formation in vivo in a mouse model. Considering these promising results, the appeal of allogeneic gene therapy products, and our evolving understanding of the importance of the immune microenvironment in bone healing, perinatal MSCs are worthy of further study.

### 5.4. Autologous vs. Allogenic Gene Therapy

Designing an ex vivo gene therapy approach for bone regeneration requires careful consideration as to whether autologous or allogeneic stem cells will be used. Each has advantages and disadvantages. For autologous cell-based gene therapy, using a patient’s own cells has the following advantages: (1) potential to collect tissue (e.g., adipose, muscle, or bone marrow) at the time of initial injury for use in a later staged reconstruction procedure and (2) immunocompatibility, which may allow implanted cells to survive longer, thereby facilitating extended transgene expression and possible engraftment of the cells into newly formed tissue. Disadvantages include the time-consuming process of ex vivo cell expansion in tissue culture, which may not be suitable for all clinical scenarios. Additionally, as was discussed in Section 5.3.1, stem cells exhibit considerable heterogeneity based on donor characteristics such as age, which may reduce the cells’ inherent therapeutic potential for some patients [175].

For allogeneic cell-based gene therapy, the greatest potential advantage is off-the-shelf availability. Cells from a healthy donor could be expanded, genetically modified, and cryopreserved for later use in multiple patients. Donor cells could be thoroughly characterized and tested for safety with standardized processes. From a manufacturing and regulatory standpoint, it would be more time- and cost-efficient to use a single donor (or batch of donors). The disadvantages of allogeneic approaches include the risk of immunogenicity, which could cause immune system activation and destruction of the implanted cells, thereby limiting therapeutic effects. Allogenic cells also have the potential for disease transmission.

Ultimately, if an allogeneic gene therapy approach is to be used, it must first be proven to be effective in preclinical models. As discussed in the preceding sections on stem cells, the potential of allogeneic gene therapy is a topic of ongoing research due to the known immunomodulatory effects of MSCs and the critical role of the immune system in bone healing [261]. Amongst MSC sources, umbilical cord, amniotic, and adipose appear to have the greatest immunomodulatory potential, making them good candidates [244]. Importantly, the studies establishing the immunomodulatory effects of various MSCs have largely been performed in vitro. Various factors such as culture conditions, passage number, transduction, and stem cell differentiation can affect immunogenicity, which require further investigation [244,262,263,264]. In vivo, allogeneic MSCs elicit an immune response. However, no studies directly compare the in vivo immune response to genetically modified MSCs from different sources and the differential effect this has on bone formation [261]. To determine an optimal allogeneic MSC candidate, in vivo studies are needed to more comprehensively characterize immune-mediated interactions with allogeneic transduced cells and the host during the bone repair process. Due to the dynamic and multifaceted nature of these interactions, leveraging new technologies such as single-cell RNA sequencing may be essential in answering these questions [265].

It is also important to keep in mind that long-term cell survival is not a requisite for therapeutic success. Allogeneic transduced cells may be able to provide substantial benefit if they are able to sufficiently avoid immune system activation for a period of days or weeks [244]. Immune system evasion may occur secondary to the inherent properties of the MSCs, as discussed above, or could be facilitated by the addition of an exogenous immunosuppressive drug. In a series of animal experiments from Huard and colleagues, they showed that immune system activation impaired bone healing in (1) immunocompetent animals treated with cells transduced by a first-generation AV vector and (2) immunocompetent animals treated with allogeneic cells (the series of studies are reviewed by Huard’s group in a recent publication [133]). Bone healing was improved when these animals were simultaneously immunosuppressed for a brief period. These results suggest that allogeneic gene therapy approaches can be augmented by a brief period of systemic immunosuppression, but further preclinical research is needed to determine optimal duration and dosing.

## 6. Scaffolds in Gene Therapy

Scaffolds have a multifaceted role in gene therapy approaches to bone tissue engineering. Central to the role of a scaffold for gene therapy is the delivery of genetically modified cells or vectors containing genetic material. In addition, an ideal scaffold would geometrically match the bone defect, provide structural support, possess structural properties to promote bone ingrowth and neovascularization, and have biodegradation characteristics that facilitate bone remodeling. Considerations include scaffold biomaterials, fabrication methods (e.g., 3D printing), and “seeding” methods to impregnate the scaffolds with cells or vector [92,266]. All of these considerations are areas of active research, a detailed discussion of which is beyond the scope of this review [61,267]. However, it is important to underscore the importance of scaffolds as a critical element for bone tissue engineering and the synergistic effect they can have for both in vivo and ex vivo gene therapy applications.

## 7. Conclusions and Future Directions

The management of segmental bone defects is a complex reconstructive challenge for orthopedic surgeons that lacks a consistently satisfactory solution across the full spectrum of injury. Current treatments include autologous bone grafting, allograft, recombinant growth factor delivery, vascularized bone grafts, induced membrane technique, and distraction osteogenesis. However, each has significant limitations, high costs, and variable success with increasing defect size.

Regional gene therapy is a strategy for bone tissue engineering that has shown considerable promise and rapid advancement in the past two decades. In particular, ex vivo gene therapy approaches provide all requisite components for bone regeneration—a population of osteoprogenitor cells, a sustained osteoinductive stimulus, and an osteoconductive scaffold. Ex vivo strategies may prove indispensable when the host environment is severely compromised and few osteoprogenitors are present. Two decades of preclinical studies have confirmed the efficacy of various in vivo and ex vivo strategies. Continued advancements are needed to fine-tune various aspects of regional gene therapy, including a safe and effective gene delivery strategy, an optimal MSC cell source, and factors related to cell expansion, transduction, and storage. With the ongoing collaborative efforts of researchers across the disciplines of bone tissue engineering, regional gene therapy is poised to soon deliver on the promise of treating the most challenging bone loss scenarios.

## Figures and Tables

**Table 3:** Properties of viral vectors.

Viral Vector	Packaging Capacity	Viral Genome	Tropism	Integration	Transduction Efficiency	Transgene Expression	Immunogenicity	Potential for Oncogenesis
**Retrovirus**	7–11 kb	ssRNA	Dividing cells only	Integrating	Moderate	Long-term	Moderate	Yes
**Lentivirus**	6–10 kb	ssRNA	Dividing and non-dividing cells	Integrating	Moderate/High	Long-term	Low/Moderate	Yes
**Adenovirus**	7–37 kb	dsDNA	Dividing and non-dividing cells	Non-Integrating	High	Transient	High	No
**Adeno-associated virus**	2–9 kb	ssDNA	Dividing and non-dividing cells	Recombinant AAV non-integrating wild type AAV may integrate	Moderate	Long-Term Transient	Low	Yes if integrating

**Table 4:** Adult and Perinatal Mesenchymal Stem Cell Sources and their Characteristics.

Cell Source	Availability	Harvest	Yield	Immunogenicity	Proliferation	Osteogenic Potential
**Bone Marrow**	+++	Invasive	++	++	++	+++
**Periosteum**	+	Invasive	+++	+ ↓ relative to BMSC	+++	+++
**Skeletal Muscle**	+++	Less invasive (e.g., biopsy)	+++	+ ↓ relative to BMSC	+++ ↑ relative to BMSC	+++
**Dental Pulp**	+	Medical procedure byproduct	+	+	+++	++ ↓ relative to BMSC
**Periodontal Ligament**	+		++	+	+++	++
**Gingival**	+		+++	+	+++	+++
**Adipose**	+++	Less invasive (e.g., liposuction)	+++	+ ↓ relative to BMSC	+++ ↑ relative to BMSC	++/+++ non-transduced: ↓ relative to BMSC BMP-2 transduced: ↑ relative to BMSC
**Skin (Fibroblast)**	+++	Less invasive (e.g., biopsy)	+++	+	++	++
**Peripheral Blood**	+++	Minimally invasive	+	+ ≈to BMSC	++	++
**Amniotic fluid**	++	Non-invasive (considered medical waste)	+	↓ relative to most other MSC sources AF<UCT/UCB<P	+++	+++
**Amnion (Placenta)**	+++		+++		+++	+++
**Umbilical Cord Tissue**	+++		+++		+++ ↑ relative to BMSC	+++
**Umbilical Cord Blood**	++		++		+++	+++

BMSC, Bone Marrow Stem Cell; ↑, higher than; ↓, lower than; ≈, similar to.
